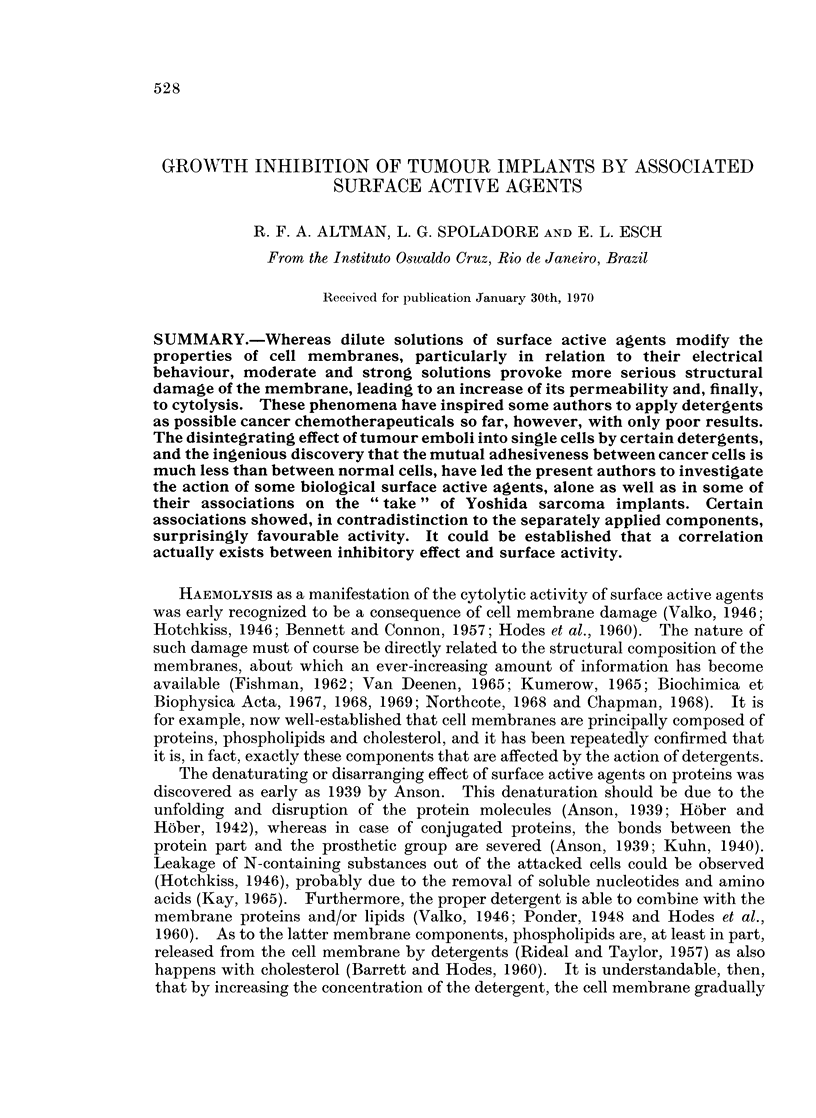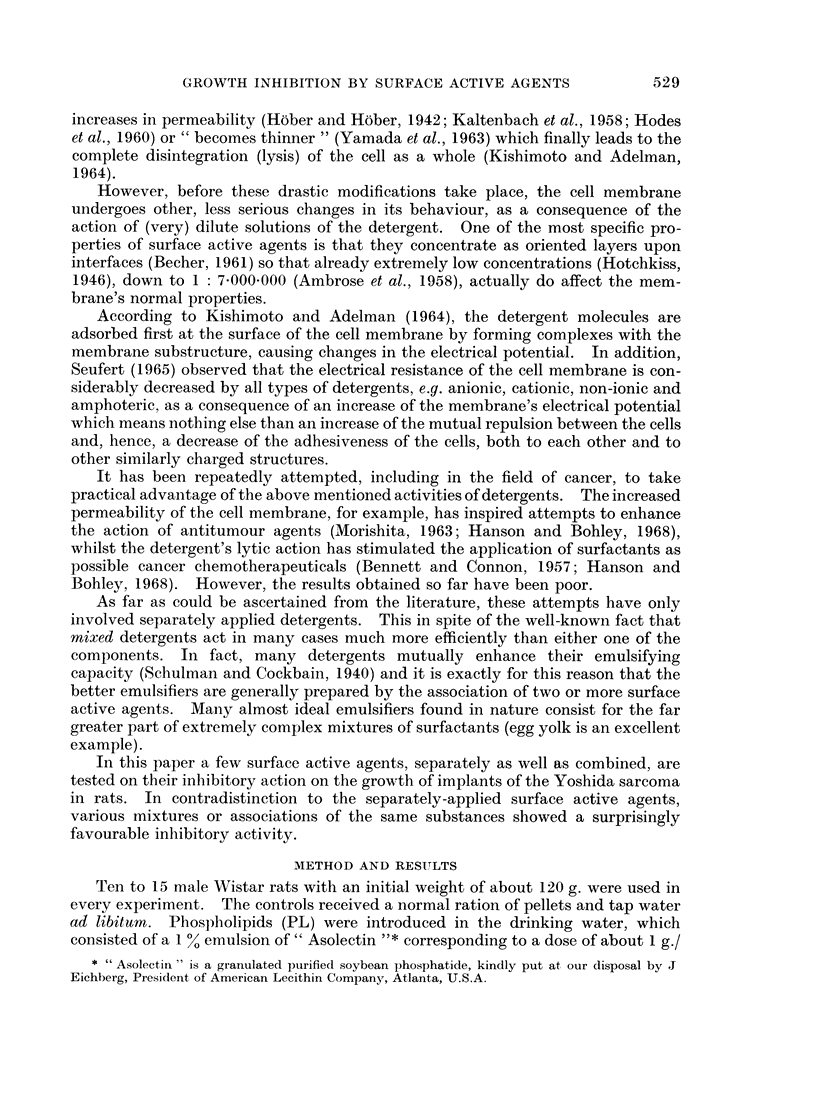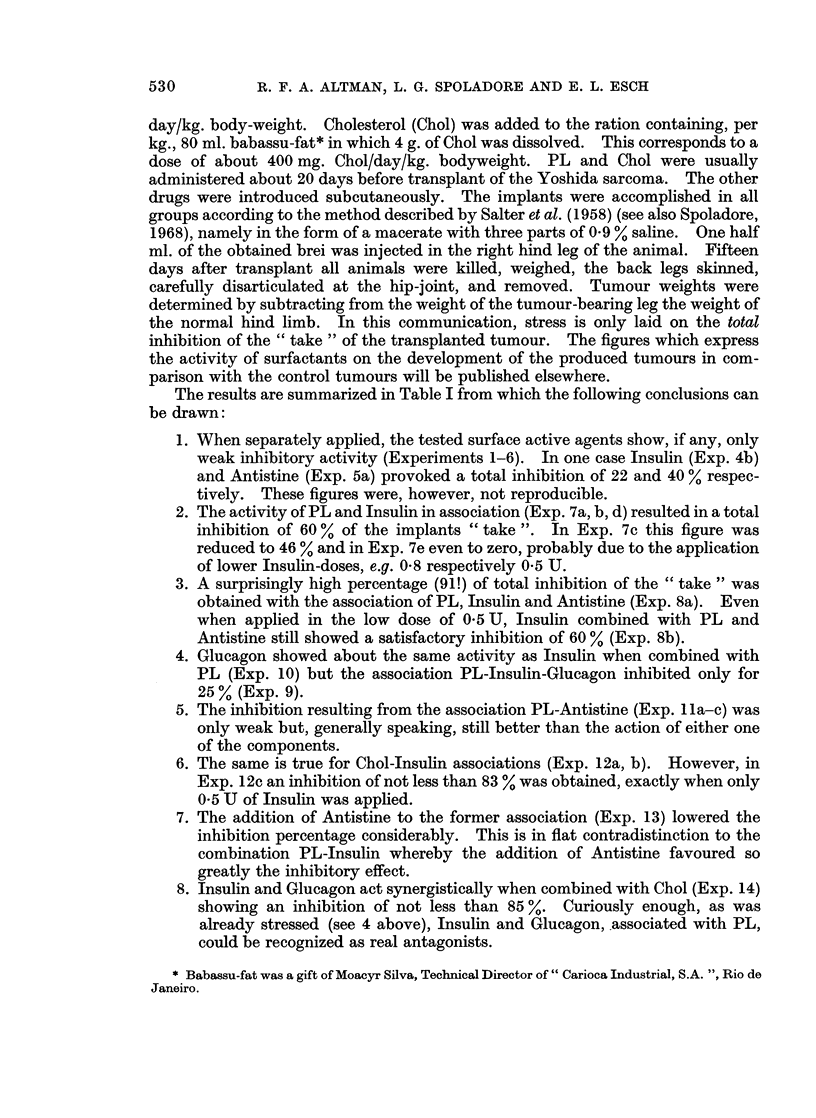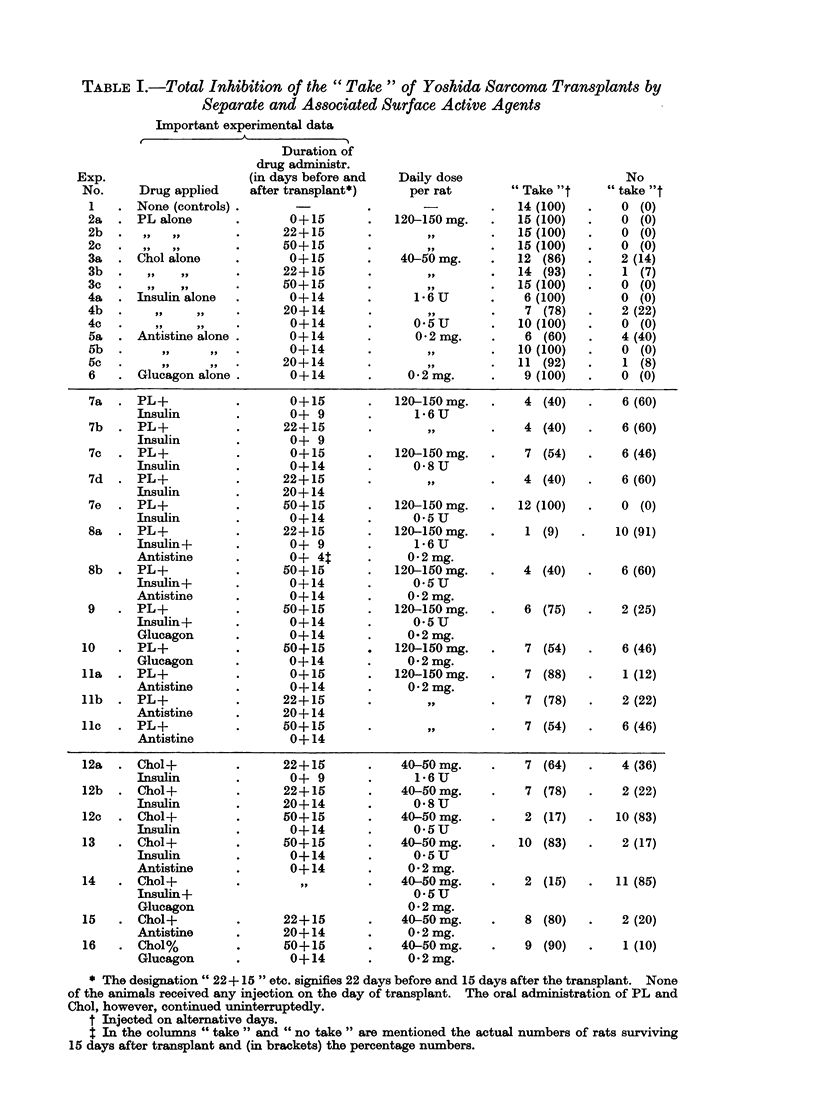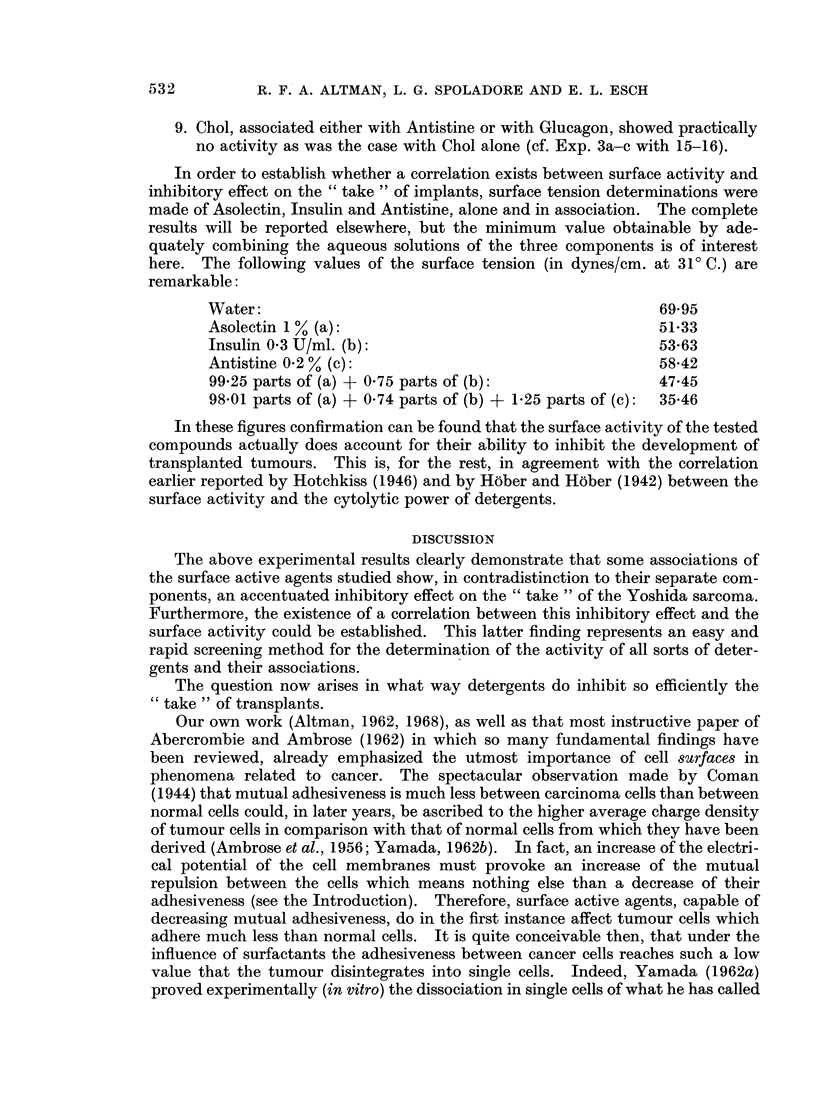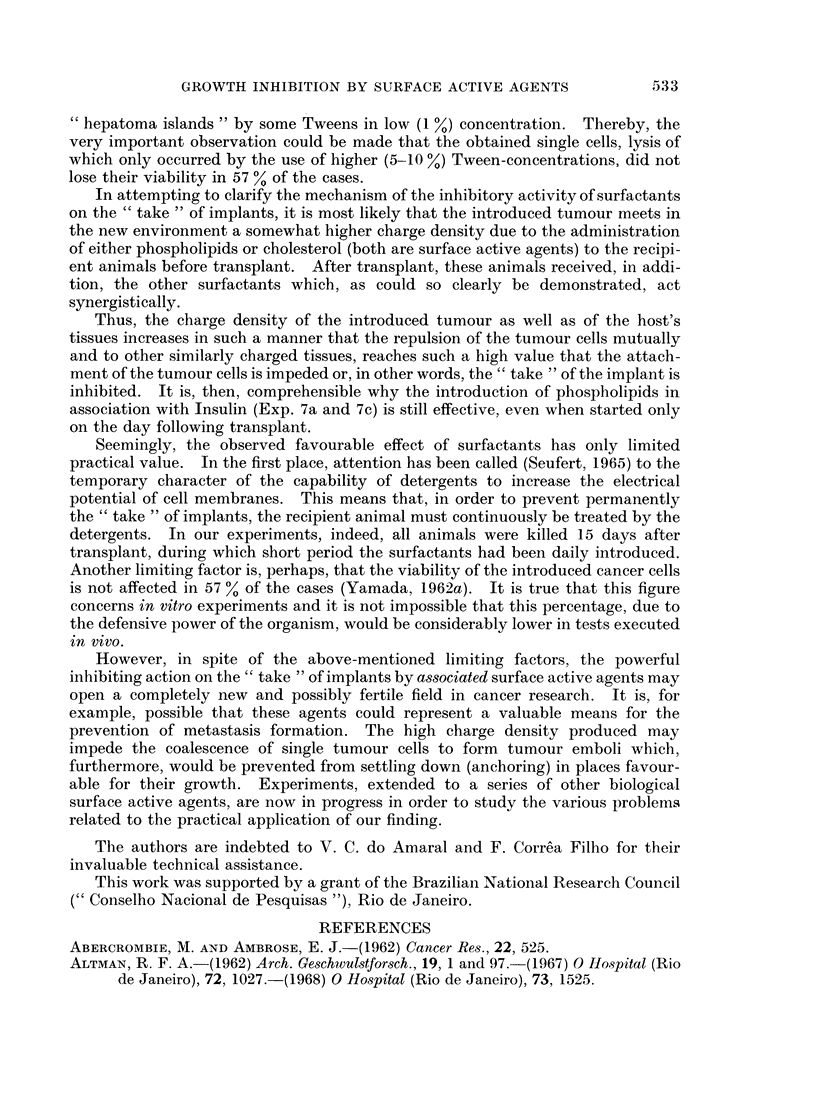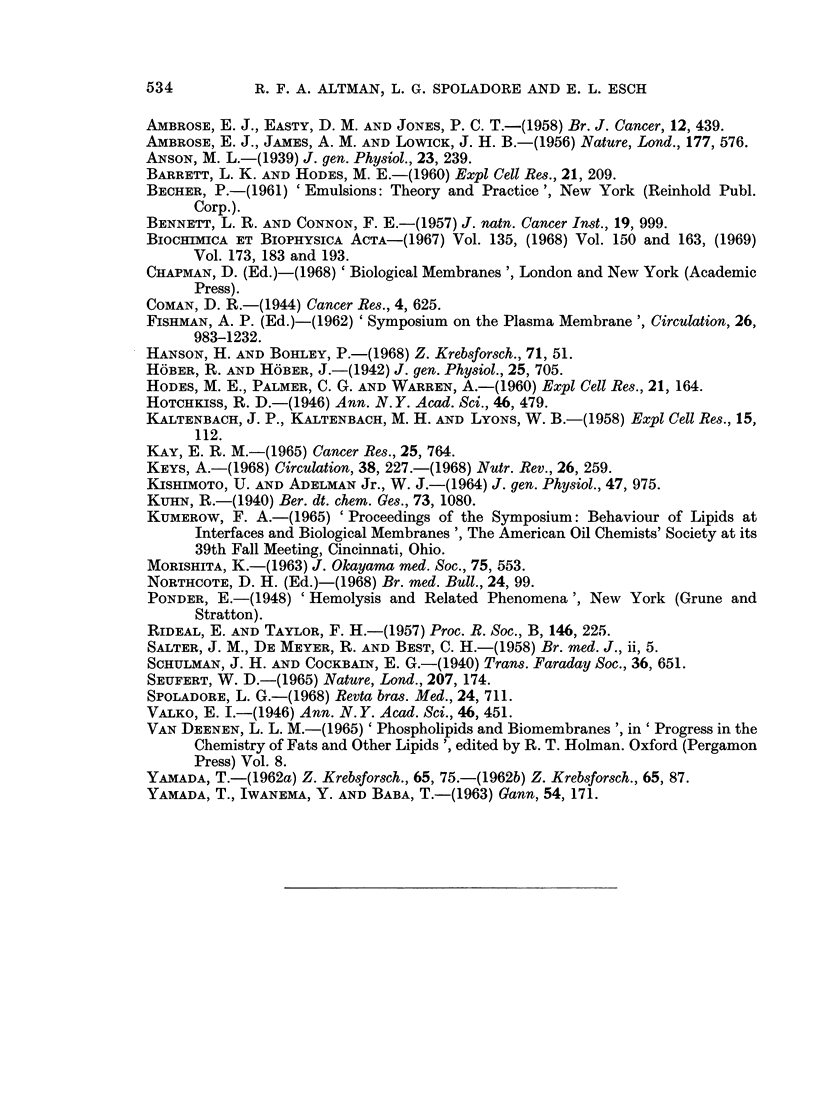# Growth Inhibition of Tumour Implants by Associated Surface Active Agents

**DOI:** 10.1038/bjc.1970.63

**Published:** 1970-09

**Authors:** R. F. A. Altman, L. G. Spoladore, E. L. Esch

## Abstract

Whereas dilute solutions of surface active agents modify the properties of cell membranes, particularly in relation to their electrical behaviour, moderate and strong solutions provoke more serious structural damage of the membrane, leading to an increase of its permeability and, finally, to cytolysis. These phenomena have inspired some authors to apply detergents as possible cancer chemotherapeuticals so far, however, with only poor results. The disintegrating effect of tumour emboli into single cells by certain detergents, and the ingenious discovery that the mutual adhesiveness between cancer cells is much less than between normal cells, have led the present authors to investigate the action of some biological surface active agents, alone as well as in some of their associations on the “take” of Yoshida sarcoma implants. Certain associations showed, in contradistinction to the separately applied components, surprisingly favourable activity. It could be established that a correlation actually exists between inhibitory effect and surface activity.


### GROWTH INHIBITION OF TUMOUR IMPLANTS BY ASSOCIATED SURFACE ACTIVE AGENTS

R. F. A. ALTMAN, L. G. SPOLADORE AND E. L. ESCH

*From the Instituto Oswaldo Cruz, Rio de Janeiro, Brazil*

Received for publication January 30th, 1970

**SUMMARY.—Whereas dilute solutions of surface active agents modify the properties of cell membranes, particularly in relation to their electrical behaviour, moderate and strong solutions provoke more serious structural damage of the membrane, leading to an increase of its permeability and, finally, to cytolysis. These phenomena have inspired some authors to apply detergents as possible cancer chemotherapeuticals so far, however, with only poor results. The disintegrating effect of tumour emboli into single cells by certain detergents, and the ingenious discovery that the mutual adhesiveness between cancer cells is much less than between normal cells, have led the present authors to investigate the action of some biological surface active agents, alone as well as in some of their associations on the " take " of Yoshida sarcoma implants. Certain associations showed, in contradistinction to the separately applied components, surprisingly favourable activity. It could be established that a correlation actually exists between inhibitory effect and surface activity.**

HAEMOLYSIS as a manifestation of the cytolytic activity of surface active agents was early recognized to be a consequence of cell membrane damage (Valko, 1946; Hotchkiss, 1946; Bennett and Connon, 1957; Hodes *et al.*, 1960). The nature of such damage must of course be directly related to the structural composition of the membranes, about which an ever-increasing amount of information has become available (Fishman, 1962; Van Deenen, 1965; Kumerow, 1965; Biochimica et Biophysica Acta, 1967, 1968, 1969; Northcote, 1968 and Chapman, 1968). It is for example, now well-established that cell membranes are principally composed of proteins, phospholipids and cholesterol, and it has been repeatedly confirmed that it is, in fact, exactly these components that are affected by the action of detergents.

The denaturating or disarranging effect of surface active agents on proteins was discovered as early as 1939 by Anson. This denaturation should be due to the unfolding and disruption of the protein molecules (Anson, 1939; Höber and Höber, 1942), whereas in case of conjugated proteins, the bonds between the protein part and the prosthetic group are severed (Anson, 1939; Kuhn, 1940). Leakage of N-containing substances out of the attacked cells could be observed (Hotchkiss, 1946), probably due to the removal of soluble nucleotides and amino acids (Kay, 1965). Furthermore, the proper detergent is able to combine with the membrane proteins and/or lipids (Valko, 1946; Ponder, 1948 and Hodes *et al.*, 1960). As to the latter membrane components, phospholipids are, at least in part, released from the cell membrane by detergents (Rideal and Taylor, 1957) as also happens with cholesterol (Barrett and Hodes, 1960). It is understandable, then, that by increasing the concentration of the detergent, the cell membrane gradually increases in permeability (Höber and Höber, 1942; Kaltenbach *et al.*, 1958; Hodes *et al.*, 1960) or " becomes thinner " (Yamada *et al.*, 1963) which finally leads to the complete disintegration (lysis) of the cell as a whole (Kishimoto and Adelman, 1964).

However, before these drastic modifications take place, the cell membrane undergoes other, less serious changes in its behaviour, as a consequence of the action of (very) dilute solutions of the detergent. One of the most specific properties of surface active agents is that they concentrate as oriented layers upon interfaces (Becher, 1961) so that already extremely low concentrations (Hotchkiss, 1946), down to 1 : 7·000·000 (Ambrose *et al.*, 1958), actually do affect the membrane's normal properties.

According to Kishimoto and Adelman (1964), the detergent molecules are adsorbed first at the surface of the cell membrane by forming complexes with the membrane substructure, causing changes in the electrical potential. In addition, Seufert (1965) observed that the electrical resistance of the cell membrane is considerably decreased by all types of detergents, *e.g.* anionic, cationic, non-ionic and amphoteric, as a consequence of an increase of the membrane's electrical potential which means nothing else than an increase of the mutual repulsion between the cells and, hence, a decrease of the adhesiveness of the cells, both to each other and to other similarly charged structures.

It has been repeatedly attempted, including in the field of cancer, to take practical advantage of the above mentioned activities of detergents. The increased permeability of the cell membrane, for example, has inspired attempts to enhance the action of antitumour agents (Morishita, 1963; Hanson and Bohley, 1968), whilst the detergent's lytic action has stimulated the application of surfactants as possible cancer chemotherapeuticals (Bennett and Connon, 1957; Hanson and Bohley, 1968). However, the results obtained so far have been poor.

As far as could be ascertained from the literature, these attempts have only involved separately applied detergents. This in spite of the well-known fact that *mixed* detergents act in many cases much more efficiently than either one of the components. In fact, many detergents mutually enhance their emulsifying capacity (Schulman and Cockbain, 1940) and it is exactly for this reason that the better emulsifiers are generally prepared by the association of two or more surface active agents. Many almost ideal emulsifiers found in nature consist for the far greater part of extremely complex mixtures of surfactants (egg yolk is an excellent example).

In this paper a few surface active agents, separately as well as combined, are tested on their inhibitory action on the growth of implants of the Yoshida sarcoma in rats. In contradistinction to the separately-applied surface active agents, various mixtures or associations of the same substances showed a surprisingly favourable inhibitory activity.

### METHOD AND RESULTS

Ten to 15 male Wistar rats with an initial weight of about 120 g. were used in every experiment. The controls received a normal ration of pellets and tap water *ad libitum*. Phospholipids (PL) were introduced in the drinking water, which consisted of a 1 % emulsion of " Asolectin "* corresponding to a dose of about 1 g./

* " Asolectin " is a granulated purified soybean phosphatide, kindly put at our disposal by J Eichberg, President of American Lecithin Company, Atlanta, U.S.A.

day/kg. body-weight. Cholesterol (Chol) was added to the ration containing, per kg., 80 ml. babassu-fat* in which 4 g. of Chol was dissolved. This corresponds to a dose of about 400 mg. Chol/day/kg. bodyweight. PL and Chol were usually administered about 20 days before transplant of the Yoshida sarcoma. The other drugs were introduced subcutaneously. The implants were accomplished in all groups according to the method described by Salter *et al.* (1958) (see also Spoladore, 1968), namely in the form of a macerate with three parts of 0·9 % saline. One half ml. of the obtained brei was injected in the right hind leg of the animal. Fifteen days after transplant all animals were killed, weighed, the back legs skinned, carefully disarticulated at the hip-joint, and removed. Tumour weights were determined by subtracting from the weight of the tumour-bearing leg the weight of the normal hind limb. In this communication, stress is only laid on the *total* inhibition of the " take " of the transplanted tumour. The figures which express the activity of surfactants on the development of the produced tumours in comparison with the control tumours will be published elsewhere.

The results are summarized in Table I from which the following conclusions can be drawn:

1. When separately applied, the tested surface active agents show, if any, only weak inhibitory activity (Experiments 1–6). In one case Insulin (Exp. 4b) and Antistine (Exp. 5a) provoked a total inhibition of 22 and 40 % respectively. These figures were, however, not reproducible.
2. The activity of PL and Insulin in association (Exp. 7a, b, d) resulted in a total inhibition of 60 % of the implants " take ". In Exp. 7c this figure was reduced to 46 % and in Exp. 7e even to zero, probably due to the application of lower Insulin-doses, *e.g.* 0·8 respectively 0·5 U.
3. A surprisingly high percentage (91!) of total inhibition of the " take " was obtained with the association of PL, Insulin and Antistine (Exp. 8a). Even when applied in the low dose of 0·5 U, Insulin combined with PL and Antistine still showed a satisfactory inhibition of 60 % (Exp. 8b).
4. Glucagon showed about the same activity as Insulin when combined with PL (Exp. 10) but the association PL-Insulin-Glucagon inhibited only for 25 % (Exp. 9).
5. The inhibition resulting from the association PL-Antistine (Exp. 11a–c) was only weak but, generally speaking, still better than the action of either one of the components.
6. The same is true for Chol-Insulin associations (Exp. 12a, b). However, in Exp. 12c an inhibition of not less than 83 % was obtained, exactly when only 0·5 U of Insulin was applied.
7. The addition of Antistine to the former association (Exp. 13) lowered the inhibition percentage considerably. This is in flat contradistinction to the combination PL-Insulin whereby the addition of Antistine favoured so greatly the inhibitory effect.
8. Insulin and Glucagon act synergistically when combined with Chol (Exp. 14) showing an inhibition of not less than 85 %. Curiously enough, as was already stressed (see 4 above), Insulin and Glucagon, associated with PL, could be recognized as real antagonists.

* Babassu-fat was a gift of Moacyr Silva, Technical Director of " Carioca Industrial, S.A. ", Rio de Janeiro.

TABLE I.—*Total Inhibition of the " Take " of Yoshida Sarcoma Transplants by Separate and Associated Surface Active Agents*

| Exp. No. | Important experimental data: Drug applied | Important experimental data: Duration of drug administr. (in days before and after transplant*) | Daily dose per rat | " Take "† | No " take "† |
|---|---|---|---|---|---|
| 1 | None (controls) | — | — | 14 (100) | 0 (0) |
| 2a | PL alone | 0+15 | 120–150 mg. | 15 (100) | 0 (0) |
| 2b | ,, ,, | 22+15 | ,, | 15 (100) | 0 (0) |
| 2c | ,, ,, | 50+15 | ,, | 15 (100) | 0 (0) |
| 3a | Chol alone | 0+15 | 40–50 mg. | 12 (86) | 2 (14) |
| 3b | ,, ,, | 22+15 | ,, | 14 (93) | 1 (7) |
| 3c | ,, ,, | 50+15 | ,, | 15 (100) | 0 (0) |
| 4a | Insulin alone | 0+14 | 1·6 U | 6 (100) | 0 (0) |
| 4b | ,, ,, | 20+14 | ,, | 7 (78) | 2 (22) |
| 4c | ,, ,, | 0+14 | 0·5 U | 10 (100) | 0 (0) |
| 5a | Antistine alone | 0+14 | 0·2 mg. | 6 (60) | 4 (40) |
| 5b | ,, ,, | 0+14 | ,, | 10 (100) | 0 (0) |
| 5c | ,, ,, | 20+14 | ,, | 11 (92) | 1 (8) |
| 6 | Glucagon alone | 0+14 | 0·2 mg. | 9 (100) | 0 (0) |
| 7a | PL+ | 0+15 | 120–150 mg. | 4 (40) | 6 (60) |
| | Insulin | 0+ 9 | 1·6 U | | |
| 7b | PL+ | 22+15 | ,, | 4 (40) | 6 (60) |
| | Insulin | 0+ 9 | | | |
| 7c | PL+ | 0+15 | 120–150 mg. | 7 (54) | 6 (46) |
| | Insulin | 0+14 | 0·8 U | | |
| 7d | PL+ | 22+15 | ,, | 4 (40) | 6 (60) |
| | Insulin | 20+14 | | | |
| 7e | PL+ | 50+15 | 120–150 mg. | 12 (100) | 0 (0) |
| | Insulin | 0+14 | 0·5 U | | |
| 8a | PL+ | 22+15 | 120–150 mg. | 1 (9) | 10 (91) |
| | Insulin+ | 0+ 9 | 1·6 U | | |
| | Antistine | 0+ 4‡ | 0·2 mg. | | |
| 8b | PL+ | 50+15 | 120–150 mg. | 4 (40) | 6 (60) |
| | Insulin+ | 0+14 | 0·5 U | | |
| | Antistine | 0+14 | 0·2 mg. | | |
| 9 | PL+ | 50+15 | 120–150 mg. | 6 (75) | 2 (25) |
| | Insulin+ | 0+14 | 0·5 U | | |
| | Glucagon | 0+14 | 0·2 mg. | | |
| 10 | PL+ | 50+15 | 120–150 mg. | 7 (54) | 6 (46) |
| | Glucagon | 0+14 | 0·2 mg. | | |
| 11a | PL+ | 0+15 | 120–150 mg. | 7 (88) | 1 (12) |
| | Antistine | 0+14 | 0·2 mg. | | |
| 11b | PL+ | 22+15 | ,, | 7 (78) | 2 (22) |
| | Antistine | 20+14 | | | |
| 11c | PL+ | 50+15 | ,, | 7 (54) | 6 (46) |
| | Antistine | 0+14 | | | |
| 12a | Chol+ | 22+15 | 40–50 mg. | 7 (64) | 4 (36) |
| | Insulin | 0+ 9 | 1·6 U | | |
| 12b | Chol+ | 22+15 | 40–50 mg. | 7 (78) | 2 (22) |
| | Insulin | 20+14 | 0·8 U | | |
| 12c | Chol+ | 50+15 | 40–50 mg. | 2 (17) | 10 (83) |
| | Insulin | 0+14 | 0·5 U | | |
| 13 | Chol+ | 50+15 | 40–50 mg. | 10 (83) | 2 (17) |
| | Insulin | 0+14 | 0·5 U | | |
| | Antistine | 0+14 | 0·2 mg. | | |
| 14 | Chol+ | ,, | 40–50 mg. | 2 (15) | 11 (85) |
| | Insulin+ | | 0·5 U | | |
| | Glucagon | | 0·2 mg. | | |
| 15 | Chol+ | 22+15 | 40–50 mg. | 8 (80) | 2 (20) |
| | Antistine | 20+14 | 0·2 mg. | | |
| 16 | Chol% | 50+15 | 40–50 mg. | 9 (90) | 1 (10) |
| | Glucagon | 0+14 | 0·2 mg. | | |

* The designation " 22+15 " etc. signifies 22 days before and 15 days after the transplant. None of the animals received any injection on the day of transplant. The oral administration of PL and Chol, however, continued uninterruptedly.

† Injected on alternative days.

‡ In the columns " take " and " no take " are mentioned the actual numbers of rats surviving 15 days after transplant and (in brackets) the percentage numbers.

9. Chol, associated either with Antistine or with Glucagon, showed practically no activity as was the case with Chol alone (cf. Exp. 3a–c with 15–16).

In order to establish whether a correlation exists between surface activity and inhibitory effect on the " take " of implants, surface tension determinations were made of Asolectin, Insulin and Antistine, alone and in association. The complete results will be reported elsewhere, but the minimum value obtainable by adequately combining the aqueous solutions of the three components is of interest here. The following values of the surface tension (in dynes/cm. at 31° C.) are remarkable:

| | |
|---|---|
| Water: | 69·95 |
| Asolectin 1 % (a): | 51·33 |
| Insulin 0·3 U/ml. (b): | 53·63 |
| Antistine 0·2 % (c): | 58·42 |
| 99·25 parts of (a) + 0·75 parts of (b): | 47·45 |
| 98·01 parts of (a) + 0·74 parts of (b) + 1·25 parts of (c): | 35·46 |

In these figures confirmation can be found that the surface activity of the tested compounds actually does account for their ability to inhibit the development of transplanted tumours. This is, for the rest, in agreement with the correlation earlier reported by Hotchkiss (1946) and by Höber and Höber (1942) between the surface activity and the cytolytic power of detergents.

### DISCUSSION

The above experimental results clearly demonstrate that some associations of the surface active agents studied show, in contradistinction to their separate components, an accentuated inhibitory effect on the " take " of the Yoshida sarcoma. Furthermore, the existence of a correlation between this inhibitory effect and the surface activity could be established. This latter finding represents an easy and rapid screening method for the determination of the activity of all sorts of detergents and their associations.

The question now arises in what way detergents do inhibit so efficiently the " take " of transplants.

Our own work (Altman, 1962, 1968), as well as that most instructive paper of Abercrombie and Ambrose (1962) in which so many fundamental findings have been reviewed, already emphasized the utmost importance of cell *surfaces* in phenomena related to cancer. The spectacular observation made by Coman (1944) that mutual adhesiveness is much less between carcinoma cells than between normal cells could, in later years, be ascribed to the higher average charge density of tumour cells in comparison with that of normal cells from which they have been derived (Ambrose *et al.*, 1956; Yamada, 1962*b*). In fact, an increase of the electrical potential of the cell membranes must provoke an increase of the mutual repulsion between the cells which means nothing else than a decrease of their adhesiveness (see the Introduction). Therefore, surface active agents, capable of decreasing mutual adhesiveness, do in the first instance affect tumour cells which adhere much less than normal cells. It is quite conceivable then, that under the influence of surfactants the adhesiveness between cancer cells reaches such a low value that the tumour disintegrates into single cells. Indeed, Yamada (1962*a*) proved experimentally (*in vitro*) the dissociation in single cells of what he has called

" hepatoma islands " by some Tweens in low (1 %) concentration. Thereby, the very important observation could be made that the obtained single cells, lysis of which only occurred by the use of higher (5–10 %) Tween-concentrations, did not lose their viability in 57 % of the cases.

In attempting to clarify the mechanism of the inhibitory activity of surfactants on the " take " of implants, it is most likely that the introduced tumour meets in the new environment a somewhat higher charge density due to the administration of either phospholipids or cholesterol (both are surface active agents) to the recipient animals before transplant. After transplant, these animals received, in addition, the other surfactants which, as could so clearly be demonstrated, act synergistically.

Thus, the charge density of the introduced tumour as well as of the host's tissues increases in such a manner that the repulsion of the tumour cells mutually and to other similarly charged tissues, reaches such a high value that the attachment of the tumour cells is impeded or, in other words, the " take " of the implant is inhibited. It is, then, comprehensible why the introduction of phospholipids in association with Insulin (Exp. 7a and 7c) is still effective, even when started only on the day following transplant.

Seemingly, the observed favourable effect of surfactants has only limited practical value. In the first place, attention has been called (Seufert, 1965) to the temporary character of the capability of detergents to increase the electrical potential of cell membranes. This means that, in order to prevent permanently the " take " of implants, the recipient animal must continuously be treated by the detergents. In our experiments, indeed, all animals were killed 15 days after transplant, during which short period the surfactants had been daily introduced. Another limiting factor is, perhaps, that the viability of the introduced cancer cells is not affected in 57 % of the cases (Yamada, 1962*a*). It is true that this figure concerns *in vitro* experiments and it is not impossible that this percentage, due to the defensive power of the organism, would be considerably lower in tests executed *in vivo*.

However, in spite of the above-mentioned limiting factors, the powerful inhibiting action on the " take " of implants by *associated* surface active agents may open a completely new and possibly fertile field in cancer research. It is, for example, possible that these agents could represent a valuable means for the prevention of metastasis formation. The high charge density produced may impede the coalescence of single tumour cells to form tumour emboli which, furthermore, would be prevented from settling down (anchoring) in places favourable for their growth. Experiments, extended to a series of other biological surface active agents, are now in progress in order to study the various problems related to the practical application of our finding.

The authors are indebted to V. C. do Amaral and F. Corrêa Filho for their invaluable technical assistance.

This work was supported by a grant of the Brazilian National Research Council (" Conselho Nacional de Pesquisas "), Rio de Janeiro.

### REFERENCES

ABERCROMBIE, M. AND AMBROSE, E. J.—(1962) *Cancer Res.*, **22**, 525.

ALTMAN, R. F. A.—(1962) *Arch. Geschwulstforsch.*, **19**, 1 and 97.—(1967) *O Hospital* (Rio de Janeiro), **72**, 1027.—(1968) *O Hospital* (Rio de Janeiro), **73**, 1525.

Ambrose, E. J., Easty, D. M. and Jones, P. C. T.—(1958) *Br. J. Cancer*, **12**, 439.
Ambrose, E. J., James, A. M. and Lowick, J. H. B.—(1956) *Nature, Lond.*, **177**, 576.
Anson, M. L.—(1939) *J. gen. Physiol.*, **23**, 239.
Barrett, L. K. and Hodes, M. E.—(1960) *Expl Cell Res.*, **21**, 209.
Becher, P.—(1961) ' Emulsions: Theory and Practice ', New York (Reinhold Publ. Corp.).
Bennett, L. R. and Connon, F. E.—(1957) *J. natn. Cancer Inst.*, **19**, 999.
Biochimica et Biophysica Acta—(1967) Vol. 135, (1968) Vol. 150 and 163, (1969) Vol. 173, 183 and 193.
Chapman, D. (Ed.)—(1968) ' Biological Membranes ', London and New York (Academic Press).
Coman, D. R.—(1944) *Cancer Res.*, **4**, 625.
Fishman, A. P. (Ed.)—(1962) ' Symposium on the Plasma Membrane ', *Circulation*, **26**, 983–1232.
Hanson, H. and Bohley, P.—(1968) *Z. Krebsforsch.*, **71**, 51.
Höber, R. and Höber, J.—(1942) *J. gen. Physiol.*, **25**, 705.
Hodes, M. E., Palmer, C. G. and Warren, A.—(1960) *Expl Cell Res.*, **21**, 164.
Hotchkiss, R. D.—(1946) *Ann. N.Y. Acad. Sci.*, **46**, 479.
Kaltenbach, J. P., Kaltenbach, M. H. and Lyons, W. B.—(1958) *Expl Cell Res.*, **15**, 112.
Kay, E. R. M.—(1965) *Cancer Res.*, **25**, 764.
Keys, A.—(1968) *Circulation*, **38**, 227.—(1968) *Nutr. Rev.*, **26**, 259.
Kishimoto, U. and Adelman Jr., W. J.—(1964) *J. gen. Physiol.*, **47**, 975.
Kuhn, R.—(1940) *Ber. dt. chem. Ges.*, **73**, 1080.
Kumerow, F. A.—(1965) ' Proceedings of the Symposium: Behaviour of Lipids at Interfaces and Biological Membranes ', The American Oil Chemists' Society at its 39th Fall Meeting, Cincinnati, Ohio.
Morishita, K.—(1963) *J. Okayama med. Soc.*, **75**, 553.
Northcote, D. H. (Ed.)—(1968) *Br. med. Bull.*, **24**, 99.
Ponder, E.—(1948) ' Hemolysis and Related Phenomena ', New York (Grune and Stratton).
Rideal, E. and Taylor, F. H.—(1957) *Proc. R. Soc.*, B, **146**, 225.
Salter, J. M., De Meyer, R. and Best, C. H.—(1958) *Br. med. J.*, ii, 5.
Schulman, J. H. and Cockbain, E. G.—(1940) *Trans. Faraday Soc.*, **36**, 651.
Seufert, W. D.—(1965) *Nature, Lond.*, **207**, 174.
Spoladore, L. G.—(1968) *Revta bras. Med.*, **24**, 711.
Valko, E. I.—(1946) *Ann. N.Y. Acad. Sci.*, **46**, 451.
Van Deenen, L. L. M.—(1965) ' Phospholipids and Biomembranes ', in ' Progress in the Chemistry of Fats and Other Lipids ', edited by R. T. Holman. Oxford (Pergamon Press) Vol. 8.
Yamada, T.—(1962*a*) *Z. Krebsforsch.*, **65**, 75.—(1962*b*) *Z. Krebsforsch.*, **65**, 87.
Yamada, T., Iwanema, Y. and Baba, T.—(1963) *Gann*, **54**, 171.